# Systematic review of methods for individual patient data meta- analysis with binary outcomes

**DOI:** 10.1186/1471-2288-14-79

**Published:** 2014-06-19

**Authors:** Doneal Thomas, Sanyath Radji, Andrea Benedetti

**Affiliations:** 1 Department of Epidemiology, Biostatistics & Occupational Health, McGill University, Montreal, Canada; 2 Department of Medicine, McGill University, Montreal, Canada; 3 Respiratory Epidemiology and Clinical Research Unit, McGill University Health Centre, Montreal, Canada; 4 Department of Biostatistics at the Dalla Lana School of Public Health, University of Toronto, Toronto, Canada; 5 K-129, The Montreal Chest Institute, 3650 St. Urbain, Montreal H2X 2P4, QC Canada

**Keywords:** Individual patient data, Meta-analysis, Random effects, Systematic review, Heterogeneity, One-stage

## Abstract

**Background:**

Meta-analyses (MA) based on individual patient data (IPD) are regarded as the gold standard for meta-analyses and are becoming increasingly common, having several advantages over meta-analyses of summary statistics. These analyses are being undertaken in an increasing diversity of settings, often having a binary outcome. In a previous systematic review of articles published between 1999–2001, the statistical approach was seldom reported in sufficient detail, and the outcome was binary in 32% of the studies considered. Here, we explore statistical methods used for IPD-MA of binary outcomes only, a decade later.

**Methods:**

We selected 56 articles, published in 2011 that presented results from an individual patient data meta-analysis. Of these, 26 considered a binary outcome. Here, we review 26 IPD-MA published during 2011 to consider: the goal of the study and reason for conducting an IPD-MA, whether they obtained all the data they sought, the approach used in their analysis, for instance, a two-stage or a one stage model, and the assumption of fixed or random effects. We also investigated how heterogeneity across studies was described and how studies investigated the effects of covariates.

**Results:**

19 of the 26 IPD-MA used a one-stage approach. 9 IPD-MA used a one-stage random treatment-effect logistic regression model, allowing the treatment effect to vary across studies. Twelve IPD-MA presented some form of statistic to measure heterogeneity across studies, though these were usually calculated using two-stage approach. Subgroup analyses were undertaken in all IPD-MA that aimed to estimate a treatment effect or safety of a treatment,. Sixteen meta-analyses obtained 90% or more of the patients sought.

**Conclusion:**

Evidence from this systematic review shows that the use of binary outcomes in assessing the effects of health care problems has increased, with random effects logistic regression the most common method of analysis. Methods are still often not reported in enough detail. Results also show that heterogeneity of treatment effects is discussed in most applications.

## Background

A meta-analysis (MA) attempts to synthesize the results from various distinct studies. The goal is to summarize the evidence for a particular statistical measure of interest, such as a risk difference or odds ratio. It is an especially important tool in clinical practice and medical research, where evidence-based information is preferred [1].

Individual patient data (IPD) MA are the gold standard of meta-analysis. In an IPD-MA line-by-line patient data are collected from the relevant studies, rather than just the measure of effect as in a standard aggregate data (AD) MA. This permits researchers to define exposures and outcomes consistently across studies, and to analyze them more similarly (e.g. adjusting for the same confounders), which may minimize heterogeneity [2,3].

For IPD-MA, two broad analytic strategies (one- and two-step approaches) are possible; both preserve the clustering of subjects within studies, comparability of study arms, and both may be either fixed or random. A fixed effects analysis assumes that the estimated effect is the same across all studies, while a random effects analysis assumes that the estimated effect varies across studies due to differences in patient populations, study procedures, etc [1,4].

A two-step approach first analyzes each study separately and as identically as possible, and then uses standard meta-analytic techniques to pool the measure of interest. The well-known random effects method of Der Simonian and Laird is frequently used in the second step of a two-step IPD-MA approach [1].

One step approaches use one statistical model while accounting for the clustering among patients in the same study, to estimate an overall effect. A one step model also takes advantage of the ability to standardize elements of the analysis across studies, but offers more flexibility to explore the differences that may exist between patients in the same study as well as across studies [2,3,5]. In particular, a one-step approach allows better control of confounding by patient- and study- level covariates, improves power for detecting interactions and subgroup analyses, as well as avoids and reduces the potential for ecological bias that may occur if group level information is included in the analysis [6,7].

In conventional AD-MA, it is difficult to estimate the effects of patient-level covariates on the treatment effect [8,9]. In the context of an AD-MA, this is known as meta-regression and may use study level covariates or aggregated patient level information. Meta-regressions are prone to ecological bias, and to confounding from variables not included in the model [5,6,9] and may have limited power. IPD-MA have higher power than meta-regression to detect the effect of an interaction between covariates and treatment, and are preferable when the interest is in estimating interactions with patient-level covariates [9-11].

Importantly, IPD-MA are not prone to ecological bias if inferences about individuals are not based on aggregated data and model misspecification is evaded [6]. For these reasons, and others, IPD-MA are considered the gold standard of meta-analysis, despite the complexity and cost of collecting the data, and are published with increasing frequency [2].

Despite the many advantages, the wide range of methods used for analysis of IPD-MA and the lack of a standardized data analysis plan is a serious drawback [12,13]. A previous review of methods used in practice for IPD-MA, reviewed 44 articles published during 1999–2001, of which 14 considered a binary outcome [13]. That review found that the two-step approach was used about two-thirds of the time [13].

The aim of this systematic review is to update that report, nearly a decade later when random effects models have been well integrated into other areas of health research, are readily available in many software packages and computing power is also up to the challenge. Our objective was to investigate the statistical approach taken to analyze IPD-MA with binary outcomes. In particular, we were interested in (i) whether two-stage or one-stage approaches were more common; (ii) how heterogeneity was investigated and reported; and (iii) if a one step approach was used, were intercepts permitted to vary across primary studies considered as random.

## Methods

Eligibility criteria for included studies were articles published in 2011 that reported results of an individual patient data meta-analysis for a binary outcome and were indexed in PUBMED or Medline. We believed that this would provide a good overview of the methods currently used for analysis of IPD-MA. We performed the search in June 2012.

We searched in PUBMED and MEDLINE for articles published between January 1, 2011 and December 30, 2011. The search terms used were “meta analysis” and (“individual patient data” or “ipd” or “patient level” or “individual participant” or “integrated analysis”). The titles and abstracts of these articles were reviewed to ensure that they reported results of an IPD-MA.

For the full text review, a standardized form was filled independently by two reviewers (SR, DT). Discordant entries were resolved by a third reviewer (AB). The data we collected from each article included: the reason for performing an IPD-MA, the goal of the IPD-MA, the types of studies collected, the number of studies sought and retrieved; the number of patients sought and retrieved; the type of outcome (e.g., binary, time-to-event or continuous); the method of analysis for the primary outcome and whether the analytic approach was one-stage or two-stage; whether intercept and/or the treatment effect were allowed to vary across studies (fixed or random effects); how heterogeneity was quantified, addressed and reported; the method of analysis of covariates: whether by one- or two-stage methods; methods for study- or patient-level covariates; and, whether subgroup analyses were performed (See Additional file 1: Table S1). For this review, we have considered only those articles which used a binary outcome.

We present descriptive analyses only.

## Results

**Figure 1:**
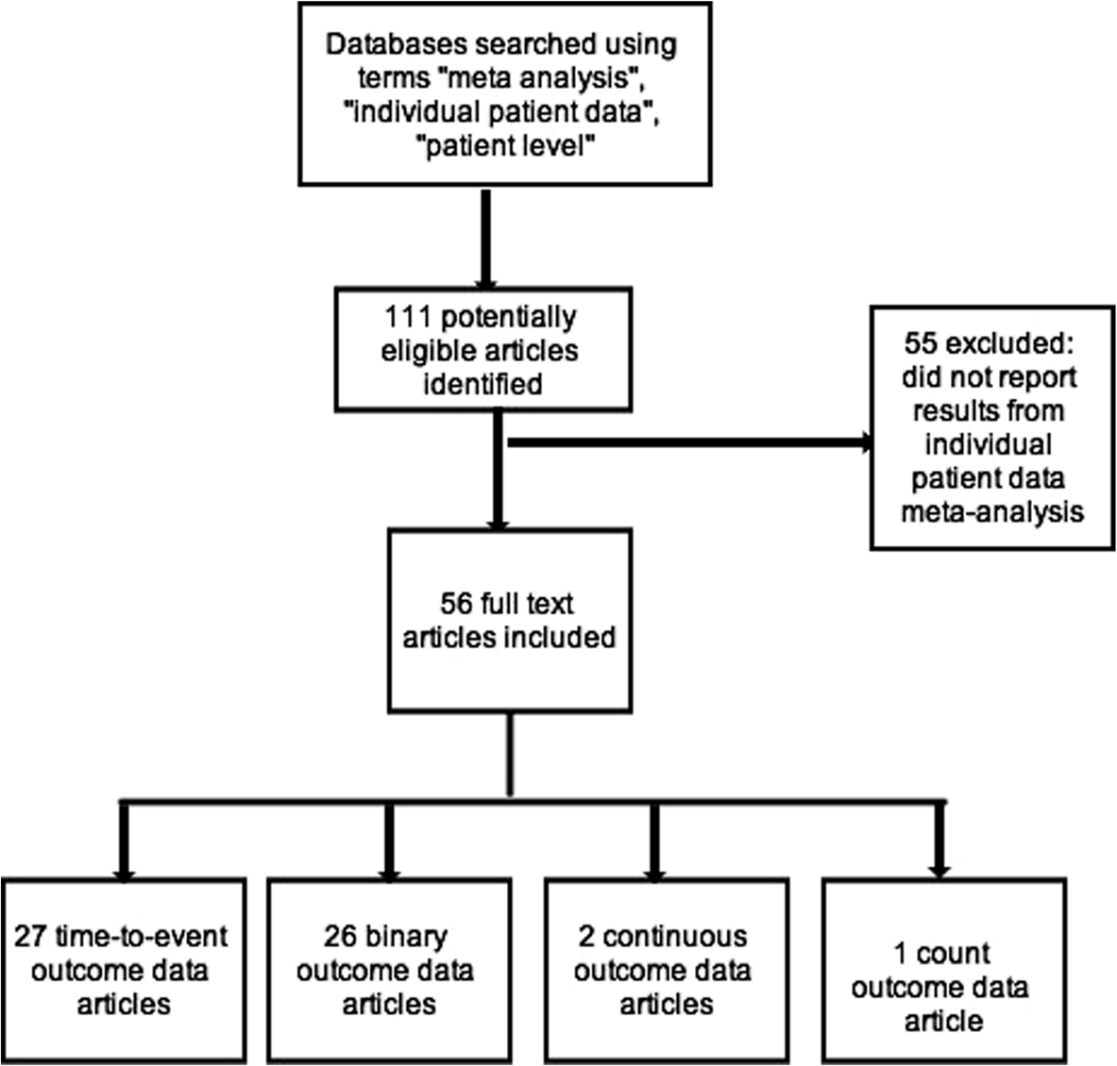
Flowchart of the inclusion of Individual patients data meta-analyses.

A total of 111 articles were returned from our search strategy. The titles and abstracts of these articles were reviewed to ensure that they reported results of an individual patient data meta-analysis. On this basis, 56 were selected for full text review. Articles excluded did not report results from an individual patient data meta-analysis (See Figure 1).

Twenty-seven articles presented time-to-event outcome data, 2 presented continuous outcome data and only one article had a count outcome. We focus on the 26 articles that presented results using a binary outcome.

Among these 26 studies, the goals of the study were to estimate diagnostic accuracy (5, 19%) [14-18]; to estimate a treatment or exposure effect (14, 53%) [19-32], to identify predictors of an outcome (4, 15%) [23,33-35], to investigate safety of a treatment (3, 12%) [32,36,37], or other reason or goal not specified (2, 8%) [38,39]. (Note that percentages may not total to 100, because more than one goal was possible) (See Table 1).

**Table 1:** **Goal of study, overall and stratified according to whether the IPD-MA included only randomized controlled trials, or included both randomized controlled trials and observational studies**^**1**^

**Reason**	**Included only randomized controlled trials (n = 15) N (%)**	**Included observational studies (n = 11) N (%)**	**Overall N (%)**
To estimate a treatment effect	10 (67%)	3 (27%)	13 (50%)
To investigate safety of a treatment	2 (13%)	1 (9%)	3 (12%)
To estimate diagnostic accuracy	1 (7%)	4 (36%)	5 (19%)
To identify predictors	1 (7%)	3 (27%)	4 (15%)
Other/Unclear	2 (13%)	1 (9%)	3 (12%)

^1^Numbers may not total to 100% because some IPD-MA had more than one goal.

Over half of IPD-MA (15/26) included only randomized control trials while the other IPD-MA included only observational studies. IPD-MA that included observational studies had a different profile in terms of goal with a greater proportion of studies that aimed to estimated diagnostic accuracy, and fewer IPD-MA that aimed to estimate the effect or safety of a treatment (See Table 1).

### Why IPD?

When carrying out an IPD-MA, there are several advantages to be gained from this approach over aggregated data meta-analyses. The main reasons for adopting the IPD method reported for these 26 articles are summarized in Table 2. Half the studies included in our review cited subgroup analyses as the reason for conducting the IPD-MA.

**Table 2:** **Reasons provided to support conducting an IPD**^**1**^

**Reason**	**N (%)**
To perform subgroup analyses	13 (50%)
To improve consistency across studies (in terms of inclusion criteria, outcome definition, etc.)	4 (15%)
To consider other outcomes	4 (15%)
To adjust for confounding variables	1 (4%)
To estimate diagnostic accuracy	5 (19%)
To identify predictors of an outcome	2 (8%)
Unclear	6 (23%)

^1^Percentages do not total to 100 because some studies reported more than one reason for conducting an IPD-MA.

### Numbers of studies and patients

**Figure 2:**
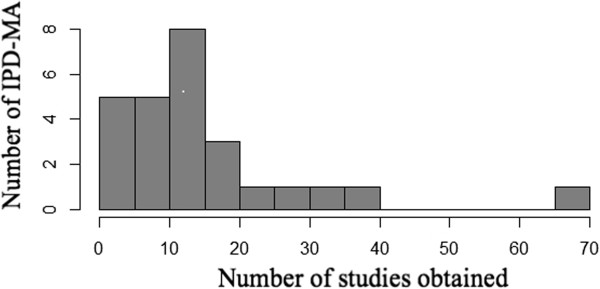
Number of studies from which IPD were obtained.

**Figure 3:**
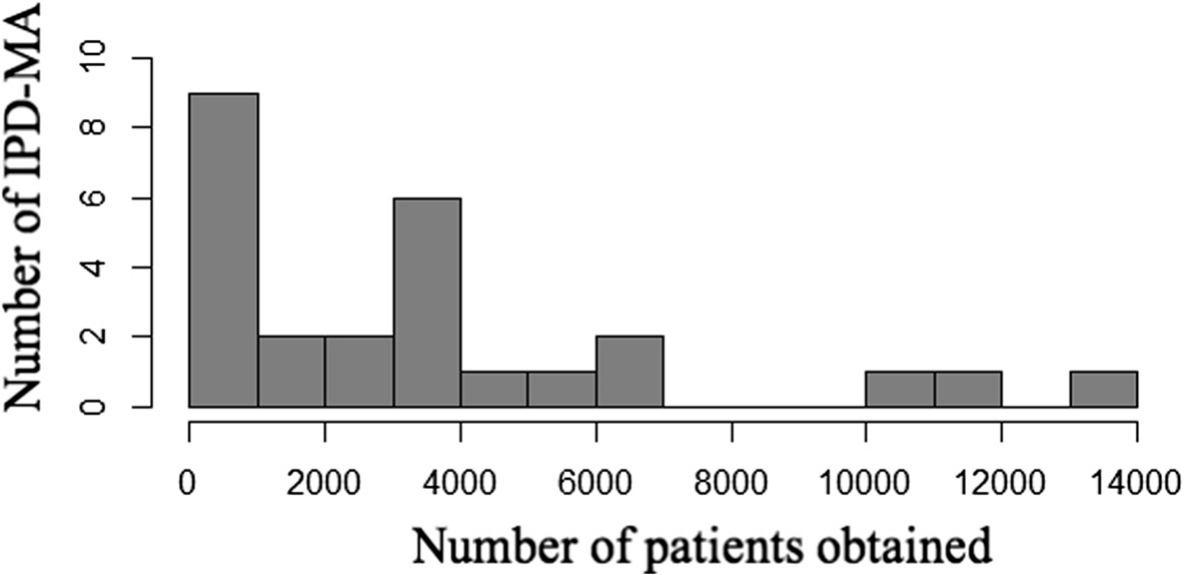
Number of patients from which IPD were obtained.

**Figure 4:**
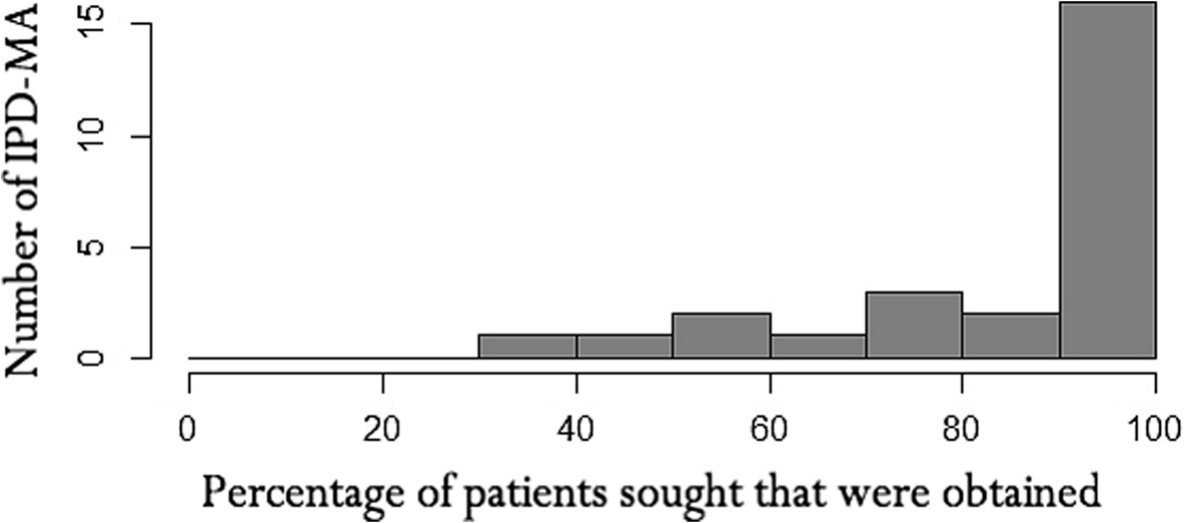
Percentage of patients sought that were obtained.

Figures 2 and 3 present the number of studies and number of patients included in the IPD-MA, respectively. More than 90% of the meta-analyses presented results for both the number of studies and patients obtained and sought. The median number of studies was 12, with inter-quartile range 6–18. The number of studies obtained in the 26 meta-analyses ranged from about ten publications with fewer than ten studies, to five with more than twenty studies.More variation was observed in the number of patients obtained, with median and inter-quartile range of 2964 and 679–4291 respectively (See Figure 3). Three meta-analyses had more than 10,000 patients and nine had fewer than 1000 patients.Figure 4 shows the percentage of patients sought for which the full data were obtained. Sixteen (62%) meta-analyses obtained 90% or more of the total number of patients. Of these, eleven (69%) publications obtained information on all of the patients sought. The median of the 16 IPD-MA was 3430 with IQR of 908–6500 patients.

### Statistical methods

Although many studies reported results for more than one outcome, here, we focus on the methods used to analyze the binary outcome. A majority of analyses concentrated on mortality or a dichotomized scale for the binary outcome. Most analyses used a one-stage method to pool the overall effect (69%) in the 26 IPD-MA for binary outcomes (Table 3). In those papers that used the one stage approach, usually all patient data from these studies were combined in a generalized linear mixed model (GLMM), accounting for the clustering among patients from the same study by including random study and or treatment effects. In general, few details were provided, and information often had to be inferred based on the results presented.

**Table 3:** Statistical analysis method categorized by overall strategy among 26 IPD meta-analyses of binary outcomes

**Analytic approach**^**1**^		**n/N (%)**
**One stage approach (n = 19)**		
Ignored clustering by study	Logistic regression	5/19 (26%)
Fixed effects	Logistic regression	4/19 (21%)
Random effects	Logistic regression	10/19 (52%)
	Fixed study effect with random treatment effect^2^	1/10 (10%)
	Random study effect with fixed treatment effect^2^	2/10 (20%)
	Random study effect with random treatment effect^2^	2/10 (20%)
	Unclear^1^	5/10 (50%)
**Two stage approach (n = 6)**		
Fixed effects	Unspecified	2/6 (33%)
	Cochrane-Mantel-Haenszel	1/6 (17%)
Random effects	Der Simonian Laird	2/6 (33%)
	Unspecified	1/6 (17%)

^1^It was unclear from one article [38] which approach was used.

^2^Among studies that used random effects logistic regression, where the intercepts and/or treatment effects allowed to vary across studies.

Among the 19 one-stage analyses, logistic regression was the most frequent technique employed. Ten of these IPD-MA used a random effects analysis. However, in 5 of these it was not clear whether intercepts, treatment effects or both were allowed to vary across studies. In the remaining 5 IPD-MA, 2 allowed both intercepts and treatment effects to vary, 1 allowed only the treatment effect to vary, and 2 allowed only the intercepts to vary. In general, little justification was offered for these choices. None specified the estimation method (e.g. penalized quasi-likelihood (PQL) [40] or adaptive Gaussian Hermite quadrature [41], etc.) used.

A fixed effects one-stage approach was used in 9 IPD-MA. Of these, 5 IPD-MA seemed to ignore clustering of subjects by study completely, and pooled all subjects together.

Two-stage methods were used in 6 of 26 studies reviewed. Of these, three studies used random effects for the treatment. One study initially used a Der Simonian Laird approach, but due to very low estimated heterogeneity, used a fixed treatment effect. The Cochrane-Mantel-Haenszel two-stage approach was used in one study, where no indication of heterogeneity across studies was found.

### Heterogeneity

Most IPD-MA (n = 20) explicitly quantified heterogeneity across included studies. (See Table 4) The most frequently used measures were the Q statistic and I^2^[42], which were used in 12 studies. In five studies, other measures of heterogeneity were reported, such as the estimated variance from the random effects model or the inclusion of an interaction term in a model. It was unclear if any measure of heterogeneity was used in 6 studies. In these studies no report or quantification of heterogeneity was presented. Two studies used multiple estimates to quantify heterogeneity; these estimates were the I^2^ and Q statistics and the Breslow-Day and Q statistic [30]. Seven studies used a one step approach but reported measures of heterogeneity based on a two-step model, while the other studies used various techniques to assess and report heterogeneity.

**Table 4:** Statistic used to measure heterogeneity among studies in the 26 IPD meta-analyses stratified by analytic approaches

	**Statistics**				
	**Q Statistics**	**I**^**2**^	**Multiple statistics**	**Other measures**	**Unclear**
	**(N = 6)**	**(N = 6)**	**(N = 2)**	**(N = 6)**	**(N = 6)**
	**n (%)**	**n (%)**	**n (%)**	**n (%)**	**n (%)**
One-step	3 (50)	4 (67)	0 (0)	6 (100)	6 (100)
Two-step	2 (33)	2 (33)	2 (100)	0 (0)	0 (0)

### Covariates

Covariates were used in three ways: (i) to assess subgroup effects; (ii) to adjust a treatment effect for possible confounders; and (iii) to identify predictors of an outcome.

Among the 16 studies where the goal of the IPD-MA was to estimate a treatment effect or the safety of a treatment, all considered subgroup analyses. Among studies that reported the number of subgroups considered, the median number of subgroups investigated was 2.5, with a range from 1–15. In all but one case, subgroups were formed by using categorical variables or categorizing a continuous variable. In one study, an interaction between the treatment and a continuous or ordinal risk score was evaluated. The subgroups investigated were based on patient-level characteristics in 13 IPD-MA, and on both patient- and study-level characteristics in 3 IPD-MA.

Among the studies that used a one-stage approach, 9/10 included interaction terms in the model, and presented stratum specific estimates as well as a p-value for the interaction. Among studies that used a two stage approach, 5/6 presented the stratum specific effect estimates, and 5/6 presented a p-value for the interaction. In two cases this p-value was calculated as described in [43].

Among the 3 IPD-MA that included observational studies and aimed to estimate a treatment effect or safety, all three adjusted for potential patient-level confounders. One of these studies used a two-step approach first adjusting for confounders in each study separately then pooling the adjusted effect estimates. Among the IPD-MA that only included randomized trials, and aimed to estimate a treatment effect or safety (n = 13), only 2 adjusted for patient level confounders. They did so by including them in a one stage model.

Finally, of the four IPD-MA that aimed to identify predictors of an outcome, three included observational studies.

### Missing data

While there are a number of approaches that could be taken to deal with missing data, 16/26 IPD-MA did not report how missing data were handled. Three studies used multiple imputation and two studies used single imputation. The remaining studies used a variety of other approaches to dealing with missing data including excluding subjects with missing data, or excluding variables with too much missing data, or it was unclear what approach was taken.

## Discussion

In this paper, we reviewed a sample of published individual patient data meta-analyses where the primary outcome was dichotomous, focusing on the statistical approach taken and results reported. To identify relevant articles in our review, we used a thorough search strategy and assessed 26 IPD MA articles published in the year 2011 that presented results for a binary outcome. It is possible that some relevant papers that reported the results of IPD MA with binary outcomes and were published in 2011 have been missed or excluded unintentionally, but these would be unlikely to differ substantially methodologically than those included. Two reviewers extracted all information independently and a third reviewer resolved conflicts. It might also be possible due to the lack of sufficient details to distinguish the methods used, that methods were incorrectly classified since the precise method used was sometimes inferred.

This review also highlighted the strengths and weaknesses of individual patient data meta-analyses (IPD-MA) where the outcome was binary. IPD-MA are clearly the gold standard of meta-analytic methods and publications featuring results from IPD-MA are growing steadily in recent years. However, there are considerable variations in the methodology employed, for instance, the use of fixed or random effects for the estimated effect measures, measures of heterogeneity and strategies used to estimate treatment effects. In many studies, the statistical aspects were not clearly reported, with insufficient details provided to distinguish the methods used. Most times, little justification was given for the approaches taken in the studies, perhaps due to the lack of specific guidelines available for the IPD meta-analysis of binary outcomes. While guidelines exist for the reporting of systematic reviews and meta-analyses, these guidelines are not specific to IPD-MA. For example, the PRISMA guideline #14 suggests that the methods of handling data and combining results, including measures of heterogeneity be described [44]. Extending those guidelines to encompass issues specific to IPD MA, such as stating if a one- or two-stage approach was used, would likely improve the reporting of IPD meta-analyses of binary outcomes.

In a previous systematic review of articles published in 1999–2001 [13], 14 (32%) of the IPD -MA dealt with a binary outcome. While the proportion was similar, we found nearly twice the number of IPD-MA of a binary outcome in just one year in 2011.

This review of 26 IPD meta-analyses of binary outcome encouragingly shows that practitioners often obtain a large proportion of the IPD required. IPD from 90% or more of the total number of studies were obtained in 62% of IPD studies, an important improvement to the 41% found in the previous review [13].

We found that more than half (73%) of studies did not use a two-step approach (i.e. analyzing each study separately and as identically as possible and pooling via standard meta analytic methods) but instead used the more flexible one-stage method. This finding was contrary to the previous review [13], in which most analyses were performed using a two-stage approach (82%) with little consideration of the one-step approach. This finding likely reflects the greater comfort with random-effects models for binary outcomes in health research, as these models are used much more frequently now and are readily available in most mainstream statistical packages.

Heterogeneity was considered in some manner by 81% of included reviews, whether by known quantitative measures or other assessments. The most frequently used measure of heterogeneity was the I^2^ statistic. Alternative measures included the Q Statistic (Chi-square statistic), and Breslow-Day test. In a few instances, heterogeneity was estimated and reported from a two-stage approach; even when a one-stage approach was used for the main analysis.

Investigating subgroup effects was one of the primary reasons for conducting an IPD-MA, and among IPD-MA that aimed to estimate a treatment effect or treatment safety all investigated subgroup effects. On the other hand, IPD-MA were unlikely to adjust for potential confounders unless observational studies were included.

Within the realm of IPD-MA with binary outcomes, our review shows that a variety of methods were used to estimate a pooled treatment effect. Many of the articles reviewed contained insufficient details on the approach used and the rationale for that approach. We next provide some recommendations and emphasize the use of the PRISMA statement to help authors ensure transparent and complete reporting of systematic reviews and meta-analyses [3,44,45]. First, if individual raw data is available for all studies and irrespective of the final approach, most statisticians and methodologists prefer the one-stage rather than a two-stage approach [2]. In some cases, the one- and two-stage approaches will give similar results [46]. However, it is currently unknown under what conditions this may be expected. Moreover, one stage methods may be preferred for evaluating treatment-covariate interactions of continuous covariates, incorporating nonlinear relationships, when studies are small, and there is heterogeneity across studies, and particularly for pooling of non randomized trials that may need to be adjusted for several confounders [46].

Moreover, methods have been developed to incorporate both individual patient data with summary level data when necessary, so that having partial IPD should not be an impediment to using a one-stage approach [5,11].

However, when random effects logistic regression is used, several details should be reported including: whether study and/or treatment were considered as random, and the statistical method used to estimate the GLMM (e.g. PQL or adaptive Gaussian Hermite quadrature). On the other hand, if a two-stage approach is used, we suggest that the meta-analytic technique used to pool results should be stated explicitly. Moreover, simply pooling subjects from various studies together is not appropriate.

Assessment and exploration of heterogeneity should always be performed in any MA, or IPD-MA. Nonetheless, how best to quantify heterogeneity remains unclear. While some advocate using the estimated variance of the random treatment effect, difficulties with its interpretation may imply that I^2^ as estimated from a two-stage approach is the optimal choice for quantifying heterogeneity. Of course, whether heterogeneity estimated from a two-stage approach is relevant to a one-stage model is an open question.There are some limitations to the work presented here. First, we have focused on binary outcomes, while survival outcomes were reported in about half of the studies retrieved (See Figure 1). Second, we limited our study retrieval to articles published in 2011. This choice was made because this gave us a sufficient sample of studies to work with that were recently completed. Moreover, we believe that there are unlikely to be major differences in the methods used, or in how they were reported between e.g. 2010 and 2011. Finally, we have focused only on the statistical approach used in these studies; whereas some may be interested more generally in how well IPD-MA are reported.

## Conclusion

As found previously, we have demonstrated that a diversity of methods are employed when dealing with IPD meta-analyses for binary outcomes. Evidence from this systematic review shows that the use IPD-MA of binary outcomes has increased, with random effects logistic regression the most common method of analysis. The statistical approach taken, along with justification for that approach, is still often not reported in sufficient detail. Standardized guidelines both for the best approach to use, as well as what details to report may be needed in this area.

## Abbreviations

IPD: Individual patient data; MA: Meta-analysis; IPD-MA: Individual patient data meta-analysis; IQR: Inter quartile range; PQL: Penalized Quasi-likelihood.

## Competing interests

The authors declare that they have no competing interests.

## Authors’ contributions

DT led this project in the writing and revision of the manuscript and was a reviewer. AB facilitated in the development of the scope, formulated the initial proposal and edited the final draft. SR contributed significantly in the screening and inclusion as a reviewer. All authors read and approved the final manuscript.

## Pre-publication history

The pre-publication history for this paper can be accessed here:

http://www.biomedcentral.com/1471-2288/14/79/prepub

## Supplementary Material

Additional file 1: Table S1 Description of the 26 IPD-MA. Click here for file
