# Supplementary material for: Systematic review of methods for individual patient data meta- analysis with binary outcomes
Source: BMC Med Res Methodol. 2014 Jun 19;14:79. doi: 10.1186/1471-2288-14-79 (PMC4074845; doi:10.1186/1471-2288-14-79)
Supplement: Additional file 1: Table S1 — Description of the 26 IPD-MA. [file 1471-2288-14-79-S1.doc]

Additional file 1: Table S1:Description of the 26 IPD-MA

| First Author | No. of trials with IPD | No. of patients with IPD | Treatment effect | Method used to estimate treatment effect | Heterogeneity |
| --- | --- | --- | --- | --- | --- |
| Askie [22] | 12 | 3298 | Fixed | One stage | Q statistic |
| Baman [28] | 18 | 2270 | Random | Two stage | I^2^ |
| Berghella [37] | 5 | 908 | Fixed | Two stage | Q statistic |
| Black [13] | 18 | 10496 | Random | One stage | Not examined |
| Bonati [23] | 3 | 3433 | Fixed | One stage | Not examined |
| Broeze [14] | 14 | 6191 | Random | One stage | I^2^ |
| Broeze [15] | 7 | 4521 | Random | One stage | Via a random intercept in model |
| Cardwell [26] | 31 | 11955 | Random | Two stage | I^2^ and Q statistic |
| De Boer [31] | 22 | 6763 | Not described -- sounds like they ignored trial | One stage | Not examined |
| Groeneveld [24] | 6 | 1119 | Fixed | One stage | I^2^ |
| He [25] | 13 | 49 | Fixed | Unclear | Q statistic |
| Houben [16] | 6 | 2787 | Random | One stage | I^2^ |
| Jefferis [35] | 3 | 622 | Fixed | Two stage | I^2^ |
| Kelder [32] | 2 | 276 | Unclear | One stage | Not examined |
| Lanas [29] | 67 | 13222 | Unclear | Two stage | Breslow day and Q statistic |
| Laporte [27] | 4 | 3600 | Random | Two stage | Q statistic |
| Lejoyeux [17] | 11 | 3354 | Random | One stage | Via random intercept in model |
| Leroy [36] | 12 | 526 | Unclear | One stage | Not examined |
| Patti [38] | 12 | 3141 | Fixed | One stage | Mantel-Haenszel method based on two stage |
| Porter [34] | 27 | 133 | Ignored study variable since all studies except one came from the same investigator, for the IPD. | One stage | Q statistic |
| Rodseth [18] | 6 | 850 | Random | One stage | I^2^ |
| Saber [30] | 12 | 5636 | Fixed | One stage | Evaluate by interaction between study and risk factor |
| Van der Pas [19] | 19 | 1168 | Random | One stage | Q statistic |
| Von [20] | 7 | 3332 | Random | One stage | Via random intercept in model |
| Waldman [33] | 14 | 223 | Not clear, seems as they ignored trial | One stage | Not examined |
| Zinkstok [21] | 36 | 180 | Random | One stage | Via random intercept in model |
